# Surgical Punctal Occlusion; Combined Lacrimal Canaliculi Cauterization and Punctal Suturing for Severe Dry Eye

**DOI:** 10.18502/jovr.v18i2.13179

**Published:** 2023-04-19

**Authors:** Kohei Kuroda, Hiroshi Toshida, Yoko Sorita, Kohei Ichikawa, Yusuke Matsuzaki, Toshihiko Ohta

**Affiliations:** ^1^Department of Ophthalmology, Juntendo University Shizuoka Hospital, Izunokuni, Shizuoka, Japan

**Keywords:** Cauterization, Dry Eye, Lacrimal Canaliculi, Lacrimal Puncta, Punctal Occlusion

## Abstract

**Purpose:**

To evaluate the treatment outcome of surgical punctal occlusion with combined canaliculi ablation and punctal suturing in patients with severe dry eye.

**Methods:**

Eleven eyes of seven patients were diagnosed with severe dry eye with decreased lacrimal secretion and were refractory to treatment with various eye drops and/or had repeatedly experienced loss of punctal plugs, and continued to experience subjective symptoms received surgical punctal occlusion. In 20 puncta, lacrimal canaliculi ablation was performed along the entire length of the lacrimal canaliculus where a diathermy needle could be inserted. After resection of the annulus fibrosus in the peri-punctal area, tight cross-stitch suturing of the puncta was performed with 8-0 absorbent thread. Visual acuity, corneal staining score according to the area (A) and density (D) classification, and Schirmer tear test (STT); tear break up time (tBUT); and subjective symptoms assessed by the University of North Carolina (UNC) and Dry Eye Management Scale were compared before and one year after surgery.

**Results:**

Recanalization occurred in 1/20 puncta (5.0% at month 5) in 1/11 eyes. Student's *t*-test showed significant improvement at one year compared with preoperative values for LogMAR value (*P* = 0.019), corneal staining score A (*P* = 0.00003) and D (*P* = 0.0003), STT (*P* = 0.004), and subjective symptoms (*P* = 0.015). No change was shown in tBUT and no serious adverse event occurred.

**Conclusion:**

This improved, minimally invasive surgical procedure has a low recanalization rate and achieves both objective and subjective improvements at one year.

##  INTRODUCTION

Dry eye disease is known not only to cause discomfort but also to be associated with keratoconjunctival epithelial injury and decreased visual acuity.^[1,2,3,4]^ Dry eye symptoms include dryness, redness, foreign body sensation, heavy sensation, pain, light sensitivity, discharge, itching, and eye fatigue with varying severity. An increasing number of treatment options are available for dry eye, including various types of eye drops and procedures for punctal closure.^[5]^ In particular, punctal plug insertion is the first choice for punctal closure because it is a simple, minimally invasive procedure.^[5,6,7,8]^

Secondary Sjogren's syndrome associated with collagen diseases such as rheumatism, cicatricial pemphigoid, and chronic Stevens–Johnson syndrome decreases lacrimal secretion, which causes dry eye.^[4,5]^ In patients with severe dry eye, subjective symptoms include eye discomfort and eye pain and keratoconjunctival epithelial impairment. Many patients are refractory to treatment, for example, they show no improvement with eye drops and repeatedly lose punctal plugs; furthermore, insertion of a punctal plug often is difficult, and granulation may form after insertion of a punctal plug.^[9]^ In such severe cases, surgical punctal closure is indicated, for example, by lacrimal canaliculi ablation.^[10,11,12,13,14,15,16,17,18,19,20,21,22,23]^

We evaluated the postoperative outcome of the surgical punctal occlusion with combined lacrimal canaliculi cauterization and punctal suturing for severe dry eye, which was a minimally invasive procedure, obtained relatively good results that were maintained even one year after surgery.

**Table 1:** Background and subjective symptoms of the patients.


Case	**Age (yr)**	**Gender**	**Background or diseases**	**Side**	**Upper/Lower**	**Follow-up months**	**LogMAR**	
				**Pre**	**Post**
1	70	Male	Depression	Right	U + L	12.1	0.097	0.000
2	38	Female	Microphthalmos	Left	U + L	14.4	1.000	0.824
3	55	Female	RA, SS	Right	U + L	12.1	0.000	–0.079
		Left	U + L	12.1	–0.079	–0.079
4	71	Male	RA	Right	U + L	14.5	1.000	0.523
		Left	U + L	14.5	0.523	0.301
5	36	Male	SJS, schizophrenia	Right	L	12.7	0.155	0.000
		Left	L	12.7	0.523	0.523
6	46	Female	SS	Right	U + L*	12.7*	–0.079	–0.176
		Left	U + L	12.7	–0.079	–0.176
7	81	Female	SS	Left	U + L	11.6	0.523	0.000
Mean ± SD	52.7 ± 15.4			12.3 ± 2.4	0.326 ± 0.413	0.151 ± 0.338
*P*-value				0.019	
	
	
RA, rheumatoid arthritis; SS, Sjögren's syndrome; SJS, Stevens–Johnson syndrome; OD, right eye; OS, left eye; U, upper; L, lower; SD, standard deviation; LogMAR; logarithm minimum angle of resolution; yr, year *A case of recanalization in lower punctum at five months after the surgery, and the additional surgery was performed by same surgical method. Data were included through the first and the second surgeries.								

**Table 2:** Objective findings of the patients.


Case	**Side**	**Fluorescein staining**				**tBUT**		**STT**		**VAS score**	
	**Adea**		**Density**							
	**Pre**	**Post**	**Pre**	**Post**	**Pre**	**Post**	**Pre**	**Post**	**Pre**	**Post**
1	Right	2	1	2	1	4	5	9	9	5	3
2	Left	3	1	3	1	4	3	14	18	10	2
3	Right	1	0	1	0	1	2	1	1	10	7
	Left	3	1	3	1	1	3	1	1	10	8
4	Right	3	1	3	1	1	1	5	7	6	1
	Left	3	1	3	1	1	1	5	9	6	1
5	Right	3	1	3	2	1	1	5	7	8	0
	Left	3	2	3	2	1	1	3	6	8	0
6	Right	3	1	3	2	2	2	1	7	10	0
	Left	1	1	1	1	3	3	1	7	10	0
7	Left	2	1	3	1	0	1	0	1	8	2
Mean ± SD		2.5 ± 0.8	1.0 ± 0.4	2.5 ± 0.8	1.2 ± 0.6	1.7 ± 1.3	2.1 ± 1.3	4.1 ± 4.3	6.6 ± 4.9	8.3 ± 1.9	2.2 ± 2.8
*P*-value		<@0.00003		<@ 0.0002		<@ 0.096		<@ 0.004		<@ 0.0002	
	
	
tBUT, tear break up time; STT, Schirmer's tear test; VAS, visual analogue scale; UNC, University of North Carolina; SD, standard deviation											

**Figure 1:**
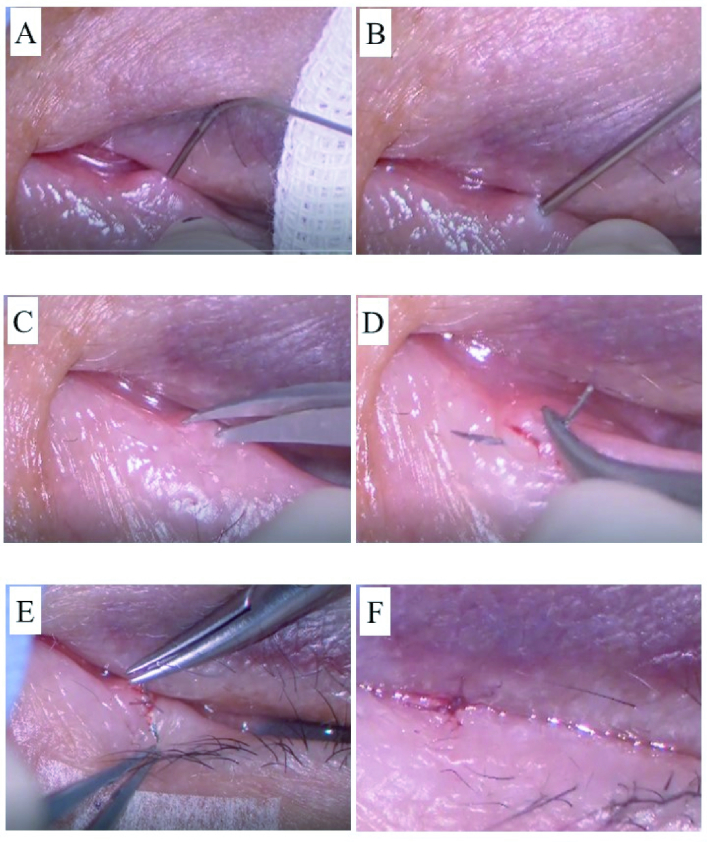
Images of surgical procedure. (A) Injection of Lidocaine into the punctum. (B) Punctal and cauterization of lacrimal canaliculi and puncta by diathermy. (C) Incision of the ring-shaped annulus fibrosus around the lacrimal punctum with a micro-scissor. (D) Suturing of lacrimal punctum with 8-0 Vicryl. (E) Second suture for cross-stitches. (F) Ligation was completed.

**Figure 2:**
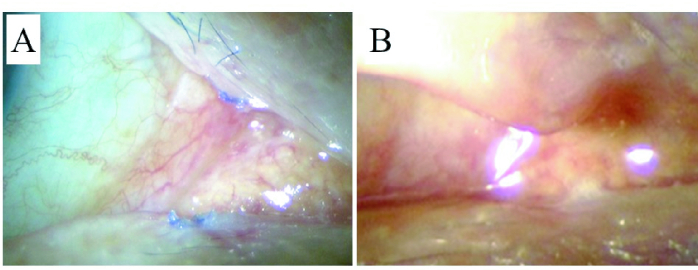
Postoperative images of case 1. Right eye of 69-year-old male. (A) Ligations were shown in both upper and lower lacrimal puncta at postoperative day 10 and were then removed. (B) After removal of suture. Lacrimal puncta were closed.

**Figure 3:**
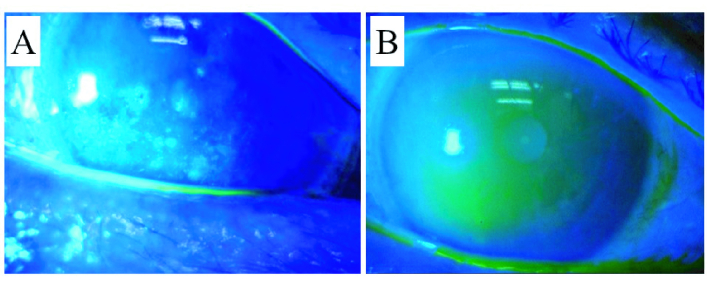
Microscopic images of case 1 after instillation of fluorescein eyedrops. (A) Fluorescein staining image prior to the surgery. The AD score was A2D2. Corrected visual acuity was 0.8 in Snellen chart. (B) Fluorescein image at postoperative day 14. No staining was shown. The AD score was A0D0. Corrected visual acuity was 1.2 in Snellen chart.

##  METHODS

### Patients

We recruited consecutive patients who visited the Department of Ophthalmology at Juntendo University Shizuoka Hospital, Shizuoka, Japan, from June 2018 through March 2021, and had a diagnosis of severe dry eye with decreased lacrimal secretion, were refractory to treatment with various eye drops, and had repeatedly experienced loss of punctal plugs. Participants were patients with a definitive diagnosis of dry eye according to the Asia Dry Eye Society diagnostic criteria,^[2]^ which include having at least one subjective symptom and a tear break-up time (tBUT) of 5s or less. The criteria for surgical intervention were being refractory to treatment with various eye drops for dry eye; loss of punctal plugs after repeated insertion; persistent subjective symptoms, such as eye pain and sense of a foreign body; and severe dry eye with decreased lacrimal secretion associated with keratoconjunctival impairment. Eleven eyes in seven patients who were followed-up for more than one year after punctal closure were included in the study.

### Procedures

The study protocol was reviewed and approved by the ethics committee of Juntendo University Shizuoka Hospital, Japan, and the approval number is 809 (approved 12.02.2021). All patients signed the informed consent form to participate in the study. Patients provided written informed consent, and the study was performed in strict adherence with the guidelines for human studies and the World Medical Association Declaration of Helsinki. First, for local anesthesia, 1% xylocaine containing epinephrine was subcutaneously injected at the peri-punctal area and directly into the punctum [Figure 1A]. A 25-gauge diathermy needle with a diameter of 0.51 mm was inserted into the canaliculi about 2 mm vertically from the punctum [Figure 1B], and similar to the insertion of the nasolacrimal bougie, the tip of the diathermy needle was directed horizontally and inserted through the canaliculi to the place where it appeared to have reached the lacrimal sac. Next, the entire length of the canaliculi epithelium (about 8–10 mm) was cauterized while the inserted diathermy needle was slowly removed. To prevent recanalization of the canaliculi and facilitate closure of the punctum by suturing, the ring-shaped annulus fibrosus around the lacrimal punctum was incised parallel to the eyelid margin with a micro-scissor [Figure 1C]. Then, the incised skin around the punctum was cauterized with a diathermy needle in the same manner as for hemostasis, and a diathermy needle was inserted to cauterize the inside and outside of the punctum again. In the final step, the punctum was sutured with cross-stitches of 8-0 Vicryl absorbent thread across the punctum [Figures 1D & 1E], and the operation was completed [Figure 1F]. After the operation, 1.5% levofloxacin hydrate ophthalmic solution was instilled four times a day for one week. The absorbent thread was removed only if damage was seen on the surface of the eye [Figure 2].

### Endpoints

The recorded data included patient characteristics, including diagnosis; corrected visual acuity before and after the operation, assessed by converting Snellen visual acuity into the Logarithm of the Minimum Angle of Resolution value (LogMAR); corneal staining score according to the AD classification,^[24]^ which semi-quantitatively determines the area (A) and density (D) of fluorescein staining on a 3-point scale; tBUT; tear secretion volume assessed by the Schirmer tear test (STT); subjective symptoms assessed by the University of North Carolina (UNC); Dry Eye Management Scale,^[25]^ intraoperative and postoperative complications; the presence or absence of recanalization after surgery; and any differences in eye drop use before and after the operation. The data were retrospectively reviewed based on the information in the medical records.

### Statistical Analysis

Results are presented as means ± standard deviations and ranges. The pre- and postoperative values of items were statistically compared by Student's paired *t*-test. A *P*-value < 0.05 was considered as statistically significant.

##  RESULTS

The study participants comprised 11 eyes of seven patients (five eyes of three men and six eyes of four women), including 20 puncta (9 upper and 11 lower puncta). The mean age at surgery was 52.4 ± 14.3 years (range, 36–81 years), and the mean follow-up period was 12.7 ± 0.8 months (range, 12.1–14.4 months) [Table 1]. The diagnoses were as follows: primary Sjogren's syndrome – two patients, three eyes; Sjogren syndrome secondary to rheumatism – one patient, two eyes; depression – one patient, one eye; schizophrenia – one patient, two eyes; and Stevens–Johnson syndrome – one patient, two eyes. Two eyes of one patient with four puncta with Sjogren syndrome comprised reoperation after recanalization by cautery of puncta [Table 2]. During the one-year postoperative follow-up period, recanalization was observed in one eye with one puncta in one patient (5.0%) five months after the operation; the operation was repeated by the same surgical method, and the second postoperative result was favorable. The findings in this patient one year after the first surgery and six months after the second surgery were aggregated.

The corrected visual acuity converted to the LogMAR value significantly improved after the operation (0.326 ± 0.423 before vs 0.168 ± 0.321 after; *P* = 0.019; paired *t*-test) [Table 2]. The corneal staining A score also significantly improved (2.4 ± 0.8 before vs 1.0 ± 0.4 after; *P* = 0.00003; paired *t*-test), as did the D score (2.5 ± 0.8 before vs 1.1 ± 0.5; *P* = 0.0003; paired *t*-test). The images of typical improved case is shown in Figure 3. STT values significantly improved (4.1 ± 4.3 mm before vs 6.6 ± 4.9 mm after; *P* = 0.004) too. No significant difference was seen in BUT (1.7 ± 1.3 s before vs 2.1 ± 1.3 s after; *P* = 0.167), but subjective symptoms showed a significant improvement (8.3 ± 1.9 before vs 2.2 ± 2.8 after; *P* = 0.015). No intraoperative complications were observed. Postoperative complications included keratitis due to suture contact with the cornea one week after surgery (9.1%) in one eye in one patient; the infiltration was healed one week later after treatment with antibacterial eye drops.

##  DISCUSSION

According to previous reports, the success rate of lacrimal canaliculi ablation for severe dry eye varies from 14% to 100% because the punctum and canaliculi can show recanalization after punctal closure; the cause of recanalization is unknown, however, it is assumed that the punctum and canaliculi are not sufficiently adhered.^[10]^ Therefore, several techniques have been developed to reduce the rate of recanalization.^[10,11,12,13,14,15,16,17,18,19,20,21,22,23]^ A 65% success rate has been reported when a square of skin is cut out at the punctal opening, rotated by 180º and reinserted so that the skin covering the punctal opening no longer has an opening.^[10]^ The success rate is 92% for canaliculi epithelial destruction by a drill and punctal suturing,^[11]^ and 100% for closing the lacrimal punctum with a patch,^[12]^ canaliculectomy,^[13]^ or transfer of lacrimal punctum to dry dock.^[14]^ However, the higher the success rate, the more invasive the surgical procedure is.^[10]^ One of the greatest drawbacks of surgical punctal closure is that it is an open, invasive operation. Other new surgical techniques include first removing and reinserting a square of skin at the punctal opening, as described above,^[10]^ and then closing the punctal opening with the semi-lunar fold of the bulbar conjunctiva. However, this approach is also relatively invasive. The aforementioned surgical techniques have the common goal to maintain the closure of the punctum so that it does not reopen. However, because all these techniques are relatively invasive, we developed an improved method for punctal closure^[23]^ in patients with severe dry eye in which we combined lacrimal ablation and punctal suturing.

This study evaluated a novel, minimally invasive approach to surgical punctal occlusion that more reliably destroyed the canaliculi epithelium by performing ablation over the entire length of the canaliculi within the range in which the diathermy needle could be inserted and by using a new suturing method. The operation achieved significant improvements in objective and subjective variables. Previous studies^[10,11,12,13,14,15,16,17,18,19,20,21]^ also performed ablation over the entire length of the canaliculi, but unlike the approach in our study, they did so with a high heat energy laser.^[21]^ In the present study, we made an incision in the annulus fibrosus around the ring-shaped lacrimal punctum, with a scalpel parallel to the eyelid margin. We sutured end to end with cross-stitches across the punctum; our aim was to perform tight suturing. As a result, recanalization was observed in only one eye in one patient at month five, and recanalization did not occur in any other eyes up to one year. This recanalization rate of 5.0% was comparable or better than that of previous reports, indicating that our technique was more effective in closing the punctum. Because we considered that the postoperative inflammatory reaction might lead to adhesion of the canaliculi destruction site, we used an absorbent thread for suturing and did not use anti-inflammatory eye drops after the operation.

The advantage of our surgical procedure is that it does not require any special equipment and can be performed with a commercially available diathermy needle, standard suture thread, and general ophthalmic surgical equipment. The disadvantage is the risk of keratitis due to suturing. In our study, keratitis occurred in one eye in the early postoperative period, but we believe that it might have been avoided if the suture thread had been kept away from the cornea.

In the group of patients who underwent surgery with this surgical technique, tBUT and Schirmer levels did not significantly improve, but corrected visual acuity and corneal staining score did. In contrast to these inconsistent results, some studies reported that all these objective variables improved. We suggest that the Schirmer level may not have improved in our study because although our surgical technique increased tear retention in the meniscus after surgery, it did not affect tear secretion itself. Subjective symptoms improved with our new surgical approach. Considering that the latest dry eye diagnostic criteria place more emphasis on the presence of subjective symptoms,^[1,2,3,4]^ this finding appears to be of great significance.

For patients with severe dry eye in whom it is difficult to insert a punctal plug or who experience repeated loss of punctal plugs, the method of punctal closure by lacrimal canaliculi cauterization and punctal suturing presented here appears to be an effective and minimally invasive treatment option. Additional studies that evaluate the long-term prognosis in larger sample sizes are warranted.

##  Financial Support and Sponsorship

This work was supported in part by a Grant-in-Aid for Special Research in Subsidies for ordinary expenses of private schools from the Promotion and Mutual Aid Corporation for Private Schools of Japan.

##  Conflicts of Interest

There are no conflicts of interest.

## References

[B1] Tsubota K, Pflugfelder SC, Liu Z, Baudouin C, Kim HM, Messmer EM, et al (2020). Defining dry eye from a clinical perspective. Int J Mol Sci.

[B2] Tsubota K, Yokoi N, Watanabe H, Dogru M, Kojima T, Yamada M, et al

[B3] Clayton JA (2018). Dry eye. N Engl J Med.

[B4] Wolffsohn JS, Arita R, Chalmers R, Djalilian A, Dogru M, Dumbleton K, et al (2017). TFOS DEWS II diagnostic methodology report. Ocul Surf.

[B5] Jones L, Downie LE, Korb D, Benitez-Del-Castillo JM, Dana R, Deng SX, et al (2017). TFOS DEWS II management and therapy report. Ocul Surf.

[B6] Ervin AM, Law A, Pucker AD (2017). Punctal occlusion for dry eye syndrome. Cochrane Database Syst Rev.

[B7] Ervin AM, Law A, Pucker AD (2019). Punctal occlusion for dry eye syndrome: Summary of a Cochrane systematic review. Br J Ophthalmol.

[B8] Chen F, Wang J, Chen W, Shen M, Xu S, Lu F (2010). Upper punctal occlusion versus lower punctal occlusion in dry eye. Invest Ophthalmol Vis Sci.

[B9] Horwath-Winter J, Thaci A, Gruber A, Boldin I (2007). Long-term retention rates and complications of silicone punctal plugs in dry eye. Am J Ophthalmol.

[B10] Geerling G, Tost FH (2008). Surgical occlusion of the lacrimal drainage system. Dev Ophthalmol.

[B11] Liu D, Sadhan Y (2002). Surgical punctal occlusion: A prospective study. Br J Ophthalmol.

[B12] Shalaby O, Rivas L, Rivas AI, Oroza MA, Murube J (2001). Comparison of 2 lacrimal punctal occlusion methods. Arch Soc Esp Oftalmol.

[B13] Putterman AM (1991). Canaliculectomy in the treatment of keratitis sicca. Ophthalmic Surg.

[B14] Murube J, Murube E (1996). Treatment of dry eye by blocking the lacrimal canaliculi. Surv Ophthalmol.

[B15] Dohlman CH (1978). Punctal occlusion in keratoconjunctivitis sicca. Ophthalmology.

[B16] Wang Y, Carreno-Galeano JT, Singh RB, Dana R, Yin J (2021). Long-term outcomes of punctal cauterization in the management of ocular surface diseases. Cornea.

[B17] Benson DR, Hemmady PB, Snyder RW (1992). Efficacy of laser punctal occlusion. Ophthalmology.

[B18] Nelson CC, Reed S (1991). Argon laser versus thermal cautery for punctal occlusion. An animal study Ophthalmic Plast Reconstr Surg.

[B19] Hutnik CM, Probst LE (1998). Argon laser punctal therapy versus thermal cautery for the treatment of aqueous deficiency dry eye syndrome. Can J Ophthalmol.

[B20] Knapp ME, Frueh BR, Nelson CC, Musch DC (1989). A comparison of two methods of punctal occlusion. Am J Ophthalmol.

[B21] Ohba E, Dogru M, Hosaka E, Yamazaki A, Asaga R, Tatematsu Y, et al (2011). Surgical punctal occlusion with a high heat-energy releasing cautery device for severe dry eye with recurrent punctal plug extrusion. Am J Ophthalmol.

[B22] Yokoi N, Komuro A, Sotozono C, Kinoshita S (2018). A new surgical approach for punctal occlusion using fibrous tissue from under the lacrimal caruncle. Clin Ophthalmol.

[B23] Yokoi N, Nishii M, Komuro A, Kinoshita S (2004). New surgical methods for punctal occlusion of severe tear-deficient dry eye and its outcome. Nippon Ganka Gakkai Zasshi.

[B24] Miyata K, Amano S, Sawa M, Nishida T (2003). A novel grading method for superficial punctate keratopathy magnitude and its correlation with corneal epithelial permeability. Arch Ophthalmol.

[B25] Grubbs J Jr, Huynh K, Tolleson-Rinehart S, Weaver MA, Williamson J, Lefebvre C, et al (2014). Instrument development of the UNC Dry Eye Management Scale. Cornea.

